# Supplementary material for: From Plankton to Primates: How VSP Sequence Diversity Shapes Voltage Sensing
Source: Int J Mol Sci. 2025 Nov 12;26(22):10963. doi: 10.3390/ijms262210963 (PMC12652588; doi:10.3390/ijms262210963)
Supplement: Supplementary file 1 [file ijms-26-10963-s001.zip › ijms-3920963-supplementary.pdf]

Supplementary Materials

Species GEVIs sequences

The underlined sequence correlates to the VSD. Color-coded sequences of chimeric constructs correlate to the species sequence.

>Japanese Rice Fish (*Oryzias latipes*)

MSVHFQPGSDAGVNGNVAKMEDAKVEIDNGKEESALPDTL

YHNIQRAITPFVMSFGFRLFGVVLIIVDFVLVIVDFSLAS

KSREVAKALEAVSLSISFFFLADVLLRVYVEGFKVYFRSK

LNIVDACVVVLTLVVTMVYTFSDLSGVSLIPRVVNFFRFL

RIIILVRVFRLAAQKKELEKVTRRMISENKRRGDPMSKGE

ELFTGVVPILVELDGDVNGHKFSVSGEGEGDATYGKLTLK

FICTTGKLPVPWPTLVTTLTYGVQCFSRYPDHMKRHDFFK

SAMPEGYVQERTIFFKDDGNYKTRAEVKFEGDTLVNRIEL

KGIDFKEDGNILGHKLEYNYNDHQVYIMADKQKNGIKANF

KIRHNIEDGGVQLADHYQQNTPIGDGPVLLPDNHYLFTTS

TLSKDPNEKRDHMVLLEFVTADGITHGMDELYK

>Takifugu (*Takifugu rubripes*)

MSSVYFDPGSDSGVNGNVAKMEDAEVTIDDGKDDSVVPDT

LYHNVRKKVKPVVTSFGFRVFGVVLILVDFVLVVVDISLY

DRSREVGDALEAVSLLISLFFLADVLLRVFVEGFQVYFSS

RLNIVDACVVAVTLVVTMIYTFSDLSGASLIPRAVSFLRF

LRIIILVRVFRLASQKKELEKVTRRMVSENKRRGDPMSKG

EELFTGVVPILVELDGDVNGHKFSVSGEGEGDATYGKLTL

KFICTTGKLPVPWPTLVTTLTYGVQCFSRYPDHMKRHDFF

KSAMPEGYVQERTIFFKDDGNYKTRAEVKFEGDTLVNRIE

LKGIDFKEDGNILGHKLEYNYNDHQVYIMADKQKNGIKAN

FKIRHNIEDGGVQLADHYQQNTPIGDGPVLLPDNHYLFTT

STLSKDPNEKRDHMVLLEFVTADGITHGMDELYK

>Finch (*Lonchura striata*)

MTAVRFEQGTPPRESSGSPTAEEATVKIDNGHDEGNGPDT

CPNATRRKIAPFVMSFGFRVFGVVLIIVDIIVVILDLAIS

ERKKGTEIPERVSLAIALFFLIDVLLRVFVEGFRNYFQSK

LNILDACIVVGTLLINLTYCFLDVDAAKEIPRMVIFLRIL

RIVILIRIFRLASQKQQLEKVTRRMVSENKRRGDPMSKGE

ELFTGVVPILVELDGDVNGHKFSVSGEGEGDATYGKLTLK

FICTTGKLPVPWPTLVTTLTYGVQCFSRYPDHMKRHDFFK

SAMPEGYVQERTIFFKDDGNYKTRAEVKFEGDTLVNRIEL

KGIDFKEDGNILGHKLEYNYNDHQVYIMADKQKNGIKANF

KIRHNIEDGGVQLADHYQQNTPIGDGPVLLPDNHYLFTTS

TLSKDPNEKRDHMVLLEFVTADGITHGMDELYK

>Mouse (*Mus musculus*)

MYGEKKSHLYLWMEHYGYDMPANIYKMYSQPSRKTDDANK

KVSVSASRTIKLNGSTGYDTNEQITLITNGSSLSYPDEIK

SASYADPISTKAYTNDSSVYDPGGASSSTTLYELNSLSEV

SKEIITQGESALLRDKEATSELKIPSTLQTQTSMSTNTLS

LSDLSSDYQEEQMNKCKLNQMSKLYDDDERTDIQKSYWNV

VKKFVRILVSSVAFRIFGIFLVILDVFLVVVDLNVSEKKI

YIPLDYRSISLAIALFFLVDILLRVSVEGRRRYFSDVLNT

LDAVVIGVTVVVAVIYALYDKHFLRDIPRLAVLLRPLRLL

ILIRILQLAHQKRQLERLTRKLVSGNKRRGDPMSKGEELF

TGVVPILVELDGDVNGHKFSVSGEGEGDATYGKLTLKFIC

TTGKLPVPWPTLVTTLTYGVQCFSRYPDHMKRHDFFKSAM

PEGYVQERTIFFKDDGNYKTRAEVKFEGDTLVNRIELKGI

DFKEDGNILGHKLEYNYNDHQVYIMADKQKNGIKANFKIR

HNIEDGGVQLADHYQQNTPIGDGPVLLPDNHYLFTTSTLS

KDPNEKRDHMVLLEFVTADGITHGMDELYK

>Giant Panda (*Ailuropoda melanoleuca*)

MIKRIVYSLASSLTFRIFGISLIFVDMSLIITDLLVTESS

MYIPLEYRSISLAIALFFFVDVLLRVYVEGIQQYFSDLLN

YLDAVIIVVTLLVDMIYMFYDFKSLQTIPRLTILFRPLRL

IILIRVFHLAHQKRHLEKLARRMVSGNKRRGDPMSKGEEL

FTGVVPILVELDGDVNGHKFSVSGEGEGDATYGKLTLKFI

CTTGKLPVPWPTLVTTLTYGVQCFSRYPDHMKRHDFFKSA

MPEGYVQERTIFFKDDGNYKTRAEVKFEGDTLVNRIELKG

IDFKEDGNILGHKLEYNYNDHQVYIMADKQKNGIKANFKI

RHNIEDGGVQLADHYQQNTPIGDGPVLLPDNHYLFTTSTL

SKDPNEKRDHMVLLEFVTADGITHGMDELYK

>Chinese hamster (*Cricetulus griseus*)

MLSGVVTAAGNKRIKEALGKPDVSTVSSGLKTVCSTVSTS

ILIASSDITSDIQEESINKSKLRQVSKFYDGDECIDIRKT

YHNTIKEVVLFLVSSVAFRVFGIMLIFLDIVFVSIDLHLV

KNQLYIPLEYRSVSFAIALFFLVDVLLRVYVEGRQQYFSD

LLNTLDAVVIGVTVFIAFTYIFYDKKFLGDNPRLAVLFRP

LWLLILVRILQLAHQKRHLEKLTRRLVSGNKRRGDPMSKG

EELFTGVVPILVELDGDVNGHKFSVSGEGEGDATYGKLTL

KFICTTGKLPVPWPTLVTTLTYGVQCFSRYPDHMKRHDFF

KSAMPEGYVQERTIFFKDDGNYKTRAEVKFEGDTLVNRIE

LKGIDFKEDGNILGHKLEYNYNDHQVYIMADKQKNGIKAN

FKIRHNIEDGGVQLADHYQQNTPIGDGPVLLPDNHYLFTT

STLSKDPNEKRDHMVLLEFVTADGITHGMDELYK

>Manatee (*Trichechus manatus latirostris*)

MSVFKSVSSGTKFILFYFDRDIRTTRNPLRGVAMDTEFLL

QYCLVSSARIVQKELTLAVCDRDIPESSKTKRIVNSIVSS

FSFRVFGVLLVFVDVSLVLTGLIFTDSSQMVSLKYRSVSL

AIASFFLVDVFLRVYVEGKRQYFSDMLNSLDAIIIVVILL

VNIIYTFHDFQGLNNIPRLAVLFRSLRLIILIRVFHLAYQ

KRHLERLTRRMVSGNKRRGDPMSKGEELFTGVVPILVELD

GDVNGHKFSVSGEGEGDATYGKLTLKFICTTGKLPVPWPT

LVTTLTYGVQCFSRYPDHMKRHDFFKSAMPEGYVQERTIF

FKDDGNYKTRAEVKFEGDTLVNRIELKGIDFKEDGNILGH

KLEYNYNDHQVYIMADKQKNGIKANFKIRHNIEDGGVQLA

DHYQQNTPIGDGPVLLPDNHYLFTTSTLSKDPNEKRDHMV

LLEFVTADGITHGMDELYK

>Human – hVSP2

MNESPDPTDLAGVIIELGPNDSPQTSEFKGATEEAPAKES

PHTSEFKGAARVSPISESVLARLSKFEVEDAENVASYDSK

IKKIVHSIVSSFAFGLFGVFLVLLDVTLILADLIFTDSKL

YIPLEYRSISLAIALFFLMDVLLRVFVERRQQYFSDLFNI

LDTAIIVILLLVDVVYIFFDIKLLRNIPRWTHLLRLLRLI

ILLRIFHLFHQKRQLEKLIRRRVSENKRRGDPMSKGEELF

TGVVPILVELDGDVNGHKFSVSGEGEGDATYGKLTLKFIC

TTGKLPVPWPTLVTTLTYGVQCFSRYPDHMKRHDFFKSAM

PEGYVQERTIFFKDDGNYKTRAEVKFEGDTLVNRIELKGI

DFKEDGNILGHKLEYNYNDHQVYIMADKQKNGIKANFKIR

HNIEDGGVQLADHYQQNTPIGDGPVLLPDNHYLFTTSTLS

KDPNEKRDHMVLLEFVTADGITHGMDELYK

>Sea Squirt (*Ciona intestinalis*)

MEGFDGSDFSPPADLVGVDGAVMRNVVDVTINGDVTAPPK

AAPRKSESVKKVHWNDVDQGPSEKPETRQEERIDIPEISG

LWWGENEHGVDDGRMEIPTTGVGRVQFRVRAVIDHLGMRV

FGVFLIFLDIILMIIDLSLPGKSESSQSFYDGMALALSCY

FMLDLGLRIFAYGPKNFFTNPWEVADGLIIVVTFVVTIFY

TVLDEYVQETGADGLGRLVVLARLLRVVRLARIFYSHQQM

KASSRRTISQNKRRGDPMSKGEELFTGVVPILVELDGDVN

GHKFSVSGEGEGDATYGKLTLKFICTTGKLPVPWPTLVTT

LTYGVQCFSRYPDHMKRHDFFKSAMPEGYVQERTIFFKDD

GNYKTRAEVKFEGDTLVNRIELKGIDFKEDGNILGHKLEY

NYNDHQVYIMADKQKNGIKANFKIRHNIEDGGVQLADHYQ

QNTPIGDGPVLLPDNHYLFTTSTLSKDPNEKRDHMVLLEF

VTADGITHGMDELYK

>Chinese sea turtle (*Pelodiscus sinensis*)

MYRFQKTEEASIKIDDGCAEDNEPDTCSRRIKKKISPFVM

SFGFRVFGVLLIFVDITLVIVDLAISDKKRSMRDTLEGIS

LAIALFFLVDVLLRVFVEGFNNYFRSKLNILDAVIVVGTL

LINMVYSFSDFSGADKIPRMVIVLRALRIIILMRILRLAS

QKKQLEKVTRRMVSENKRRGDPMSKGEELFTGVVPILVEL

DGDVNGHKFSVSGEGEGDATYGKLTLKFICTTGKLPVPWP

TLVTTLTYGVQCFSRYPDHMKRHDFFKSAMPEGYVQERTI

FFKDDGNYKTRAEVKFEGDTLVNRIELKGIDFKEDGNILG

HKLEYNYNDHQVYIMADKQKNGIKANFKIRHNIEDGGVQL

ADHYQQNTPIGDGPVLLPDNHYLFTTSTLSKDPNEKRDHM

VLLEFVTADGITHGMDELYK

>Sea hare (*Aplysia californica*)

MAAYEKFGNSDVEKGSPRNSITTADAAEITDAQIEAGEKE
EGKVTFYELENKSGVEYAADPAGDPFAPKSDFERVQRAVQ
RVIENLYFRAFTVILILLDFILVIVDLSLYSCATNDQPLE
IISHIIICYFVVEVVARIFYQGKSFLYNWLDVLDFFVVMI
SFIVDVVFMALSDSGCSGSSRYAQLVVIGRIIRIIRVVRI
VYIMIVQHRQVAKATRQMVSQNKRRGDPMSKGEELFTGVV
PILVELDGDVNGHKFSVSGEGEGDATYGKLTLKFICTTGK
LPVPWPTLVTTLTYGVQCFSRYPDHMKRHDFFKSAMPEGY
VQERTIFFKDDGNYKTRAEVKFEGDTLVNRIELKGIDFKE
DGNILGHKLEYNYNDHQVYIMADKQKNGIKANFKIRHNIE
DGGVQLADHYQQNTPIGDGPVLLPDNHYLFTTSTLSKDPN
EKRDHMVLLEFVTADGITHGMDELYK

>Honey bee mite (*Varroa destructor*)

MSYGRLDNNNDLATSSDRNNTDIPPQKGVTETKLTVEASD
LRGSSRVNCSHSNGTNAPQIDGLHHLNTKALNQIPIKHHL
LRRVVEHLAFRLVALLLIVTDICLLIVALVENPESKKLVI
YDNIALAFSVIFVLEIALRIYSLGTTDFFRKWYNKVDFAV
VMLTFIITVIEPRIEQVHTVAKAVVVGRLVRVVGFVRFLR
FYTEKNNLAKGARHVISENKRRGDPMSKGEELFTGVVPIL
VELDGDVNGHKFSVSGEGEGDATYGKLTLKFICTTGKLPV
PWPTLVTTLTYGVQCFSRYPDHMKRHDFFKSAMPEGYVQE
RTIFFKDDGNYKTRAEVKFEGDTLVNRIELKGIDFKEDGN
ILGHKLEYNYNDHQVYIMADKQKNGIKANFKIRHNIEDGG
VQLADHYQQNTPIGDGPVLLPDNHYLFTTSTLSKDPNEKR

DHMVLLEFVTADGITHGMDELYK

>Spider (*Parasteatoda tepidariorum*)

MNKYEVFENEQETESHNFKPDSEVIDVNVGKTMENSSVAI

HISKSKLVGQSLFVENSVDNGGSHVVTIQDPAEMEEVGTE

VGFMQFQLKRIVEHLIFRVFSMILIIADISILITALAMTN

KTHEQDEAFEIVAICFVAYFLFEVFIRISAKGAKGFFNDW

YNVVDLVVVVISFVVTVIYTSVDLGFGYAKLVVVGRLIRV

VGFVRLYTERKNLVKGARQMVSQNKRRGDPMSKGEELFTG

VVPILVELDGDVNGHKFSVSGEGEGDATYGKLTLKFICTT

GKLPVPWPTLVTTLTYGVQCFSRYPDHMKRHDFFKSAMPE

GYVQERTIFFKDDGNYKTRAEVKFEGDTLVNRIELKGIDF

KEDGNILGHKLEYNYNDHQVYIMADKQKNGIKANFKIRHN

IEDGGVQLADHYQQNTPIGDGPVLLPDNHYLFTTSTLSKD

PNEKRDHMVLLEFVTADGITHGMDELYK

>Plankton (*Eurytemora carolleeae*)

MERNRTTDNGNVLVDPGFVSPIYDFGEDERRKKNGLSRTR

SSSIHADPHQIEISLEEGAGDPTDEVLDRAAGFFVEHNEN

YEISWADASHMISDAETDPSKPPVPGLNKEYLTWRLRRTI

ESIFFRLFTLLLILVDIVIVIVDLSIEGSQPGLQIVDLVI

SIYFVIEVSLRLIALKPHAFFIHWYNVLDLVVILVTFIIS

VIALSGTNWAEGLSLFTALRFVRIVRFVRIYTEKKNIETA

ARQLISQNKRRGDPMSKGEELFTGVVPILVELDGDVNGHK

FSVSGEGEGDATYGKLTLKFICTTGKLPVPWPTLVTTLTY

GVQCFSRYPDHMKRHDFFKSAMPEGYVQERTIFFKDDGN

YKTRAEVKFEGDTLVNRIELKGIDFKEDGNILGHKLEYN

YNDHQVYIMADKQKNGIKANFKIRHNIEDGGVQLADHYQ

QNTPIGDGPVLLPDNHYLFTTSTLSKDPNEKRDHMVLLE

FVTADGITHGMDELYK

<SE A227D (FP domain)

MSKGEELFTGVVPILVELDGDVNGHKFSVSGEGEGDATYG

KLTLKFICTTGKLPVPWPTLVTTLTYGVQCFSRYPDHMKR

HDFFKSAMPEGYVQERTIFFKDDGNYKTRAEVKFEGDTLV

NRIELKGIDFKEDGNILGHKLEYNYNDHQVYIMADKQKNG

IKANFKIRHNIEDGGVQLADHYQQNTPIGDGPVLLPDNHY

LFTTSTLSKDPNEKRDHMVLLEFVTADGITHGMDELYK

<Finch/Human Chimera

MTAVRFEQGTPPRESSGSPTAEEATVKIDNGHDEGNGPD

TCPNATRRRIRSIVSSFAFGLFGVFLVLLDVTLILADLI

FTDSKLYIPLEYRSISLAIALFFLMDVLLRVFVERRQQY

FSDLFNILDTAIIVILLLVDVVYIFFDIKLLRNIPRWTH

LLRLLRLIILLRIFHLFHQKRQLEKLIRRRVSENKRRYR

MSKGEELFTGVVPILVELDGDVNGHKFSVSGEGEGDATY

GKLTLKFICTTGKLPVPWPTLVTTLTYGVQCFSRYPDHM

KRHDFFKSAMPEGYVQERTIFFKDDGNYKTRAEVKFEGD

TLVNRIELKGIDFKEDGNILGHKLEYNYNDHQVYIMADK

QKNGIKANFKIRHNIEDGGVQLADHYQQNTPIGDGPVLL

PDNHYLFTTSTLSKDPNEKRDHMVLLEFVTADGITHGMD

ELYK

<Human/Finch Chimera

MNESPDPTDLAGVIIELGPNDSPQTSEFKGATEEAPAKE

SPHTSEFKGAARVSPISESVLARLSKFEVEDAENVASYD

SKIKKRIRFVMSFGFRVFGVVLIIVDIIVVILDLAISER

KKGTEIPERVSLAIALFFLIDVLLRVFVEGFRNYFQSKL

NILDACIVVGTLLINLTYCFLDVDAAKEIPRMVIFLRIL

RIVILIRIFRLASQKQQLEKVTRRMVSENKRRYRMSKGE

ELFTGVVPILVELDGDVNGHKFSVSGEGEGDATYGKLTL

KFICTTGKLPVPWPTLVTTLTYGVQCFSRYPDHMKRHDF

FKSAMPEGYVQERTIFFKDDGNYKTRAEVKFEGDTLVNR

IELKGIDFKEDGNILGHKLEYNYNDHQVYIMADKQKNGI

KANFKIRHNIEDGGVQLADHYQQNTPIGDGPVLLPDNHY

LFTTSTLSKDPNEKRDHMVLLEFVTADGITHGMDELYK
